# Sedentary and Physically Active Behavior Patterns Among Low-Income African-American and White Adults Living in the Southeastern United States

**DOI:** 10.1371/journal.pone.0059975

**Published:** 2013-04-03

**Authors:** Sarah S. Cohen, Charles E. Matthews, Lisa B. Signorello, David G. Schlundt, William J. Blot, Maciej S. Buchowski

**Affiliations:** 1 International Epidemiology Institute, Rockville, Maryland, United States of America; 2 Division of Cancer Epidemiology and Genetics, Nutritional Epidemiology Branch, National Cancer Institute, Rockville, Maryland, United States of America; 3 Department of Epidemiology, Harvard School of Public Health, Boston, Massachusetts, United States of America; 4 Department of Psychology, Vanderbilt University, Nashville, Tennessee, United States of America; 5 Division of Epidemiology, Department of Medicine, Vanderbilt Epidemiology Center, Vanderbilt University School of Medicine; and the Vanderbilt-Ingram Cancer Center, Nashville, Tennessee, United States of America; 6 Division of Gastroenterology, Hepatology and Nutrition, Department of Medicine, Vanderbilt University School of Medicine, Nashville, Tennessee, United States of America; Pennington Biomedical Research Center, United States of America

## Abstract

Increased sedentary behavior and lack of physical activity are associated with increased risk for many chronic diseases. Differences in leisure-time physical activity between African American and white adults have been suggested to partially explain racial disparities in chronic disease outcomes, but expanding the definition of physical activity to include household and occupational activities may reduce or even eliminate racial differences in total physical activity. The objective of this study was to describe patterns of active and sedentary behaviors in black and white adults and to examine these behaviors across demographic measures. Sedentary and physically active behaviors were obtained from a validated physical activity questionnaire in 23,021 black men, 9,899 white men, 32,214 black women, and 15,425 white women (age 40–79) at enrollment into the Southern Community Cohort Study. Descriptive statistics for sedentary time; light, moderate, and vigorous household/occupational activity; sports/exercise; total activity; and meeting current physical activity recommendations via sports/exercise were examined for each race-sex group. Adjusted means were calculated using multiple linear regression models across demographic measures. Study participants spent approximately 60% of waking time in sedentary behaviors. Blacks reported more television viewing time than whites (45 minutes for females, 15 minutes for males), but when sitting time was expressed as a proportion of overall awake time, minimal racial differences were found. Patterns of light, moderate, and vigorous household/occupational activity were similar in all race/sex groups. 2008 Physical Activity Guidelines for Americans were followed by 16% of women and 25% of men independent of race. Overall, black and white men and women in this study spent the majority of their daily time in sedentary behaviors and less than one-fourth followed current guidelines for physical activity. These results indicate that public health campaigns should focus on both reducing sedentary behavior and increasing physical activity in all adult US populations.

## Introduction

Physically active as well as sedentary behaviors occur in many settings including at home, work, as part of transportation, and in leisure-time. Low levels of physical activity have consistently been associated with increased risk of all-cause mortality [1], [2] and many chronic diseases, including cardiovascular disease [1], diabetes [3], and certain cancers [4], [5]. Additionally, growing evidence indicates that increased sedentary time may also increase mortality risk independently from time spent in active behaviors [6]–[9]. Less time spent in leisure-time physical activity has been routinely reported for African Americans compared with whites in the United States [10]–[15], and thus, it may be that differences in these behaviors contribute to racial disparities in a range of disease outcomes that are associated with physical activity levels. However, some studies have also noted that differences in activity levels are diminished or even reversed when the definition of physical activity is broadened beyond leisure-time activities to also include activities done at home and work [12], [14]. There is a need to evaluate the full spectrum of active and sedentary behaviors in large populations of middle-age and older black and white adults in order to gain a better understanding of racial differences in these behaviors that may contribute to the disparate incidence and mortality rates of many chronic diseases.

The Southern Community Cohort Study (SCCS) is an ongoing prospective cohort study assessing disparities in cancer and other chronic disease risk. The SCCS provides an excellent opportunity to evaluate the patterns of activity-related behaviors in both black and white adults of similar socioeconomic status (SES) within the southeastern United States. Thus, the objective of this report was to describe patterns of active and sedentary behaviors in black and white adults enrolled in the SCCS and to examine associations with these behaviors by age, sex, and SES measures.

## Materials and Methods

### Ethics Statement

All participants provided written informed consent administered by trained study interviewers, and protocols were approved by Institutional Review Boards at Vanderbilt University and Meharry Medical College.

### Study Population and Design

**Figure 1:**
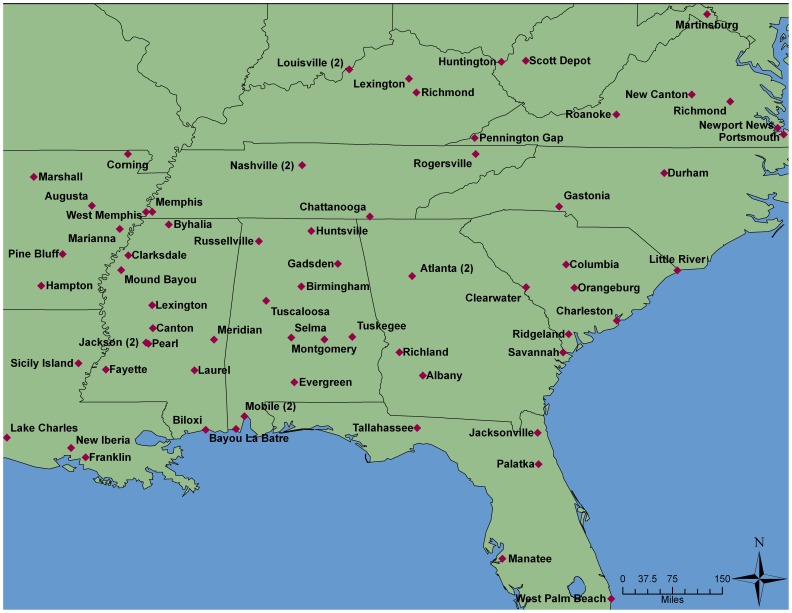
Locations of Participating Community Health Centers, the Southern Community Cohort Study.

The SCCS is an ongoing prospective cohort study in 12 states in the southeastern United States [16], [17]. Nearly 86,000 adults were enrolled in the cohort from 2002–2009. Most participants (86%) enrolled in-person at one of 71 participating federally-qualified community health centers (CHCs) (Figure 1), institutions which provide basic health and preventative services mainly to low income and uninsured persons. Enrollees included patients of the CHC, individuals accompanying patients, those utilizing other services of the CHC (e.g. pharmacy or dental services), and individuals who came to the CHC specifically to enroll in the study. Participant enrollment was on average 977 participants per CHC (range 82–4,718). The remaining 14% of the cohort was enrolled in 2004–2006 by responding to a mailed questionnaire sent to randomly selected residents of the same 12 states. SCCS eligibility requirements included being age 40–79 years, ability to speak English, and not being under treatment for cancer (with the exception of non-melanoma skin cancer) within the past 12 months. The study enrollment protocol was designed so that approximately two-thirds of enrollees would be black.

### Data Collection

Participants completed a baseline survey at enrollment that was administered by a trained study interviewer in-person for CHC-enrollees and using a self-administered questionnaire for mail-enrollees. The CHC interviews were conducted using laptop computers and a specially designed computer-assisted personal interview (CAPI), with substantial logic- and error-checking and skip pattern features built into the instrument. The survey, available online [18], included a detailed physical activity questionnaire (PAQ).

### Physical Activity Questionnaire (PAQ)

This instrument was developed for the SCCS and was designed to comprehensively assess both active and sedentary behaviors done at home, work, and during leisure-time (sports/exercise). Five questions about sedentary behaviors asked for the amount of time per day spent sitting: (a) in a car or bus, (b) at work, (c) watching television or seeing movies, (d) using a computer at home, and (e) for other reasons (e.g., sitting at meals, talking on the phone, reading, playing games, or sewing). Time spent in light, moderate, and hard (vigorous) activity at home and work were assessed separately for week- and weekend days. Examples of these light (e.g., standing at work, light office work, shopping, cooking, child care), moderate (e.g., manufacturing work, cleaning house, gardening, mowing lawn, home repair), and vigorous activities (e.g., loading trucks, construction work, farming) were provided to participants using hand cards. In terms of sports and exercise participation, information about time spent per week in moderate (e.g., dancing, golf, softball) and vigorous (e.g., jogging, aerobics, weight lifting, basketball) activities were also obtained. In addition, two global walking questions asked about time spent in slow (e.g., moving around the house, walking at work, walking the dog) and fast walking (climbing stairs, walking for transportation, or for exercise). Since the walking questions encompassed all activity domains, results from these questions were scored and evaluated separately from the other items on the questionnaire. The PAQ also asked about time spent sleeping (hrs/d) on weekend and week days.

The SCCS PAQ was evaluated in 118 participants (87 black, 31 white) using test-retest reliability methodology (assessed via Spearman correlations between PAQs administered upon study entry and then again 12–15 months later) [19]. Criterion validity of the PAQ was assessed via comparison to a physical activity monitor (accelerometer) and a last-month physical activity survey (LMPAS), administered up to four times in the study period [19]. The PAQ test-retest reliability ranged from 0.25–0.54 for sedentary behaviors and 0.22–0.47 for active behaviors. The criterion validity for the PAQ compared to PA monitor ranged from 0.21–0.24 for sedentary behaviors and from 0.17–0.31 for active behaviors. Importantly, there was general consistency in the magnitude of correlations between blacks and whites in the evaluation study.

For this analysis, information that was obtained separately for both week- and weekend days was summed and averaged using weights to reflect the per day activity over the week. Durations of the active behaviors (hrs/day) were converted to estimates of physical activity energy expenditure using methods described in the Compendium of Physical Activities [20], [21], and metabolic equivalent (MET) estimates were computed for the specific activity intensity categories. The specific MET values applied to each active behavior were as follows: light household/occupational activity (2.3); moderate household/occupational activity (4.0); vigorous household/occupational activity (6.0); moderate sports/exercise (5.0); vigorous sports/exercise (8.0); slow walking (2.5); fast walking (3.5).

Quality control procedures were implemented to identify and minimize the influence of possible out of range values, while maintaining our overall objective to rank order (or “index”) individuals by their level of behavior. First, each question was range-checked (0–20 hours per day). Second, values that exceeded the 99^th^ percentile of the distribution for each survey item were trimmed to the 99^th^ percentile value for each item using the distribution of responses for the entire cohort. Next, a summary variable was created for total activity (sum of all household and occupational activities and all sports and exercise, MET-hrs/day). We employed the method of Tukey [22] which empirically defines an out of range value as one that exceeds the sum of the 75^th^ percentile, plus 1.5 times the interquartile range, for the distribution of interest. Total activity values that were defined as out of range by this method (1.6% of the values) were set to missing for all analyses.

Participants were classified as having met current physical activity recommendations via sports/exercise if they reported 150 min/week or more of moderate; 75 min/week or more of vigorous; or, 150 min/week or more of moderate and vigorous sports/exercise combined based on the activity recommendations from the *2008 Physical Activity Guidelines for Americans* [23]. The SCCS PAQ did not explicitly ask about the length of specific activity bouts, and thus it was not clear that the 10+ minute bout threshold in the current US national guidelines would be met for moderate or vigorous household and occupational activity. Therefore, we estimated the prevalence of meeting the PA recommendations using only the exercise and sports questions with the assumption that the bout length threshold was met for these activity behaviors. Sedentary time was summarized as two variables: 1) total sitting time was calculated by summing all reported sedentary time (hrs/d), and 2) proportion of awake time spent sitting was calculated by dividing total sedentary time (hrs/d) by the sum of all sedentary and active time (i.e. household and occupational activity plus sports and exercise, [hrs/d]) reported in the PAQ.

### Statistical Methods

For the present analyses, all cohort members were considered eligible (N = 84,706). After excluding 4,147 participants who reported their race as other than ‘White’ or ‘Black/African-American’, the final study population included 80,559 individuals (9,899 white males; 23,021 black males; 15,425 white females; and 32,214 black females). Our outcomes of primary of interest were the individual and summary indices of active and sedentary behaviors, and we described differences in these behaviors by sex, race, and demographic characteristics. Thus, we present results in terms of mean and SD (or standard errors), and frequency and percentages. We employed linear regression models to determine adjusted mean values of summary activity and sedentary time measures across race groups, while adjusting for known or suspected confounders including age, education, household income, employment status, body mass index (BMI), cigarette smoking, marital status, and health conditions (hypertension, diabetes, heart attack/coronary artery disease, stroke, and emphysema).

## Results

### Demographic Characteristics

The average age of participants at enrollment into the SCCS was 52.6 years (range 40–79) (Table 1). The prevalence of obesity (BMI ≥30 kg/m^2^) was 34% and 29% in white and black men, respectively, and 46% and 58% in white and black women, respectively. With the exception of white males, nearly one-third of the participants had less than a high school education and the proportion with a college degree was low. Approximately 40% of participants were working at the time of the interview and the majority of participants reported household incomes of less than $15,000 annually. Cigarette smoking was common in all groups, with prevalence reports ranging from 36% (white females) to 57% (black males). The prevalence of chronic health conditions was relatively high in all race and sex groups.

**Table 1:** Demographics of Southern Community Cohort Study participants, by gender and race, mean (SD) or frequency [%].

	Male				Female			
	White(N = 9,899)		Black(N = 23,021)		White(N = 15,425)		Black(N = 32,214)	
**Age at enrollment (yrs)**	54.6	(9.2)	51.3	(8.2)	54.3	(9.1)	52.2	(8.8)
**Body Mass Index (kg/m^2^)**	28.7	(6.2)	27.7	(6.0)	30.6	(8.0)	32.5	(8.0)
**Source of enrollment**								
Community Health Center	6675	[67]	20958	[91]	12471	[81]	29348	[91]
General Population mailing	3224	[33]	2063	[9]	2954	[19]	2866	[9]
**County of residence**								
Rural	2309	[23]	4677	[20]	3776	[24]	8325	[26]
Urban	7586	[77]	18329	[80]	11644	[76]	23885	[74]
**Education**								
<9 years	833	[8.4]	2,017	[8.8]	1,160	[7.5]	2,354	[7.3]
9–11 years	1,352	[13.7]	5,676	[24.7]	2,586	[16.8]	7,297	[22.7]
High school	3,462	[35.0]	9,397	[40.9]	5,993	[38.9]	12,459	[38.7]
Some college/junior college	1,896	[19.2]	3,942	[17.1]	3,158	[20.5]	6,558	[20.4]
College+	2,344	[23.7]	1,968	[8.6]	2,519	[16.3]	3,513	[10.9]
**Annual Household Income ($)**								
<15,000	4,058	[41.6]	13,619	[59.8]	7,488	[49.4]	18,859	[59.4]
15,000–24.9,000	1,657	[17.0]	4,860	[21.4]	2,952	[19.5]	7,299	[23.0]
25,000–49.9,000	1,690	[17.3]	2,878	[12.6]	2,492	[16.4]	4,031	[12.7]
≥50,000	2,344	[24.0]	1,403	[6.2]	2,220	[14.7]	1,565	[4.9]
**Marital status**								
Married/living with partner	5,170	[53.5]	7,283	[31.8]	6,896	[45.2]	8,625	[26.9]
Separated/divorced	2,785	[28.8]	7,633	[33.3]	5,164	[33.8]	11,074	[34.5]
Widowed	349	[3.6]	836	[3.7]	2,027	[13.3]	4,513	[14.1]
Single	1,365	[14.1]	7,152	[31.2]	1,173	[7.7]	7,843	[24.5]
**Currently Working**	4,086	[41.6]	8,810	[38.6]	5,884	[38.9]	12,749	[40.5]
**Cigarette Smoking**								
Current	4,009	[41.1]	13,008	[56.9]	5,473	[35.7]	10,468	[32.7]
Former	3,269	[33.5]	4,750	[20.8]	3,910	[25.5]	6,269	[19.6]
Never	2,480	[25.4]	5,097	[22.3]	5,933	[38.7]	15,251	[47.7]
**Health Conditions (yes)** 1								
Hypertension	4,856	[49.1]	11,815	[51.4]	7,699	[50.1]	20,565	[64.0]
Diabetes	1,788	[18.1]	4,151	[18.1]	3,108	[20.2]	7,945	[24.7]
Heart attack/coronary artery disease	1,386	[14.0]	1,496	[6.5]	1,087	[7.1]	1,677	[5.2]
Stroke/TIA	718	[7.3]	1,330	[5.8]	1,090	[7.1]	2,178	[6.8]
Emphysema	1,179	[11.9]	1,299	[5.7]	2,655	[17.3]	2,929	[9.1]
**Sleeping (hrs/day)**	7.15	(2.17)	7.46	(2.55)	7.07	(2.04)	7.31	(2.52)

1 Has a doctor ever told you that you have had… ?

### Time Spent in Sedentary Behaviors

Participants reported an average of approximately 9 hours/day, or 60% of waking time, in sedentary behavior (Table 2). Television and movie viewing was the single largest source of sitting time but only accounted for about half of all time in reported sedentary behaviors. Black women reported an average of 45 minutes more total sitting time than white women while black men reported 15 minutes more on average than white men. When sitting time was expressed as a proportion of overall time reported, there were minimal differences between blacks and whites (Table 2).

**Table 2:** Time in sedentary behaviors [mean (std) or percentage], by sex and race.

	Male				Female			
	White		Black		White		Black	
**Sedentary behaviors (hrs/day)**								
Sitting in a car or bus	1.50	(1.79)	1.74	(2.06)	1.14	(1.38)	1.36	(1.61)
Sitting at work	1.57	(2.65)	1.23	(2.42)	1.38	(2.51)	1.39	(2.52)
Watching television or movies	3.43	(2.79)	4.10	(3.09)	3.32	(2.69)	3.94	(2.99)
Using a home computer	0.65	(1.25)	0.31	(0.94)	0.66	(1.26)	0.47	(1.15)
Other sitting^1^	1.99	(1.89)	2.01	(1.88)	2.36	(2.02)	2.43	(2.06)
Total sedentary time	9.14	(4.90)	9.40	(5.36)	8.85	(4.51)	9.60	(5.22)
**Sedentary behaviors (% of awake time)** 2	61%		60%		59%		60%	

1 Examples include sitting at meals, talking on the phone, reading, playing cards, or sewing.

2 Total awake time includes all sedentary behaviors; light, moderate, and vigorous household and occupational activity; and moderate and vigorous sports and exercise.

### Time Spent in Physically Active Behaviors

Participants reported spending most of their active time in light and moderate activities (Table 3). Women reported more light activity than men (3.4 and 2.7 hours/day, respectively) but less vigorous activity (0.4 hours/day for women and 1.4 hours/day for men). Light, moderate, and vigorous household and occupational activity estimates were similar across race groups for men and women. The proportions of participants reporting any sports and exercise as well as those meeting current recommendations for physical activity were similar across race groups in both men and women. Participants reported slow walking on average 3 hours/day and fast walking for 1 hour/day with patterns similar across all race and sex groupings.

**Table 3:** Physical activity behaviors and walking [mean (std) or percentage], by gender and race.

	Male							Female							
Activity Type	White			Black				White				Black			
**Household/occupational**															
Light (hrs/d)	2.63	(2.50)		2.72	(2.60)			3.45	(2.71)			3.44	(2.70)		
Moderate (hrs/d)	2.17	(2.24)		2.41	(2.35)			2.28	(2.00)			2.41	(2.03)		
Vigorous (hrs/d)	1.20	(2.21)		1.47	(2.50)			0.47	(1.30)			0.44	(1.31)		
Total (MET-hrs/d)^1^	21.92	(20.59)		24.71	(22.47)			19.87	(15.51)			20.18	(15.51)		
**Sports and exercise**															
Sports/exercise (% reporting any)^2^	35%			37%				26%				28%			
Sports/exercise among those reporting any sports and exercise time (MET-hrs/d)	4.47		(3.76)	4.37			(4.02)	3.73			(3.34)	3.05			(3.04)
% Meeting recommendations via sports and exercise^3^	25%			25%				17%				15%			
**Overall Activity (MET-hrs/d)** 4	23.45		(21.2)	26.34		(23.40)		20.82		(15.98)		21.01		(16.01)	
**Walking**															
Slow (hrs/d)	2.99		(2.77)	3.23		(2.93)		3.11		(2.80)		3.02		(2.80)	
Fast (hrs/d)	0.98		(1.70)	1.30		(2.07)		0.90		(1.65)		1.01		(1.82)	
Total (MET-hrs/d)	10.88		(9.73)	12.57		(11.00)		10.89		(9.58)		11.03		(10.12)	

1 Calculated by multiplying time spent in each activity intensity level by metabolic equivalent estimates (METs).

2 Includes moderate and vigorous sports/exercise.

3 Current physical activity recommendations are met if participant reported 150 or more min/week of moderate sports/exercise, 75 or more min/week of vigorous sports/exercise, or 150 or more min/week of moderate and vigorous sports/exercise combined.

4 Includes household and occupational activity plus sports/exercise.

### Association of Sedentary Behaviors and Demographics

Age was inversely associated with total time spent in sedentary behaviors, but positively associated with sedentary time as a proportion of overall wake time (Tables 4 and 5). Among men and women, education was positively associated with absolute sitting time in both blacks and whites. Among women, education was also positively associated with the proportion of awake time spent sitting, but this association was less consistent among males. Higher household income was associated with increased sedentary time in all participants. Also, in all groups, participants who were currently working spent a lower proportion of their awake time in sedentary behaviors. High BMI was associated with sedentary time only among whites. Former smokers and those who never smoked reported less sitting time than did current smokers. Among males, married participants reported slightly longer sitting time and higher proportion of wake time spent sitting than those who were divorced, single, or widowed, while for females, sitting time was lowest among married participants.

**Table 4:** Sedentary behaviors among males [adjusted mean (std err)] across demographic characteristics and body mass index, by race.

	Total sitting(hrs/d)				Sitting as proportion of total wake time (%)^2^			
	White		Black		White		Black	
**Age**								
40–49	9.8	+/−0.2	10.8	+/−0.1	62.3	+/−0.8	64.6	+/−0.6
50–59	9.9	+/−0.2	10.5	+/−0.1	65.5	+/−0.8	68.1	+/−0.6
60–69	9.4	+/−0.2	10.2	+/−0.2	64.9	+/−0.8	70.5	+/−0.6
70–79	8.8	+/−0.3	9.5	+/−0.2	66.2	+/−1.1	71.1	+/−1.0
**Education**								
<9 years	8.9	+/−0.3	9.5	+/−0.2	64.0	+/−1.1	69.5	+/−0.7
9–11 years	9.5	+/−0.2	10.0	+/−0.1	63.5	+/−0.9	67.6	+/−0.6
High school	9.4	+/−0.2	10.3	+/−0.1	61.8	+/−0.8	67.5	+/−0.6
Some college/junior college	9.6	+/−0.2	10.7	+/−0.2	64.4	+/−0.8	67.7	+/−0.6
College degree and beyond	9.9	+/−0.2	10.9	+/−0.2	69.9	+/−0.9	70.5	+/−0.7
**Household Income ($)**								
<15 K	9.1	+/−0.2	9.4	+/−0.1	67.5	+/−0.8	67.7	+/−0.5
15–24.9 K	9.2	+/−0.2	10.2	+/−0.1	61.9	+/−0.9	67.1	+/−0.6
25–49.9 K	9.6	+/−0.2	10.5	+/−0.2	63.1	+/−0.9	67.5	+/−0.7
≥50 K	10.0	+/−0.2	11.0	+/−0.2	66.5	+/−0.9	72.0	+/−0.8
**Currently Working**								
Yes	9.8	+/−0.2	10.5	+/−0.1	58.8	+/−0.8	61.2	+/−0.6
No	9.1	+/−0.2	10.0	+/−0.1	70.7	+/−0.7	75.9	+/−0.6
**Body Mass Index (kg/m** 2 **)**								
<18.5	8.8	+/−0.6	10.3	+/−0.3	60.5	+/−2.4	72.2	+/−1.4
18.5–24.9	8.8	+/−0.2	9.7	+/−0.1	63.2	+/−0.7	66.4	+/−0.5
25–29.9	9.2	+/−0.1	10.0	+/−0.1	64.1	+/−0.6	66.8	+/−0.5
30–34.9	9.5	+/−0.2	10.4	+/−0.1	66.2	+/−0.7	68.1	+/−0.6
35+	11.0	+/−0.2	10.9	+/−0.2	69.7	+/−0.8	69.4	+/−0.6
**Smoking Status**								
Current	9.8	+/−0.2	10.5	+/−0.1	63.6	+/−0.8	67.6	+/−0.6
Former	9.3	+/−0.2	10.2	+/−0.1	64.8	+/−0.8	69.0	+/−0.6
Never	9.3	+/−0.2	10.1	+/−0.1	65.8	+/−0.8	69.1	+/−0.6
**Marital Status**								
Married	9.7	+/−0.2	10.5	+/−0.1	66.6	+/−0.7	69.2	+/−0.5
Separated/Divorced	9.5	+/−0.2	10.2	+/−0.1	63.7	+/−0.8	68.2	+/−0.6
Single (Never married)	9.4	+/−0.2	10.0	+/−0.1	64.8	+/−0.9	67.8	+/−0.6
Widowed	9.3	+/−0.3	10.4	+/−0.2	63.8	+/−1.3	69.0	+/−0.9

1 Includes total/occupational activity plus sports/exercise.

2 Wake time includes sitting, household/occupational activity, and sports.

NOTE: All models are adjusted for the other demographic characteristics shown in the table as well as for hypertension (Y/N), diabetes (Y/N), heart attack/coronary artery disease (Y/N), stroke/TIA (Y/N), and emphysema (Y/N).

**Table 5:** Sedentary behaviors among females [adjusted mean (std err)] across demographic characteristics and body mass index, by race**.**

	Total sitting(hrs/d)				Sitting as proportion of total wake time (%)^2^			
	White		Black		White		Black	
**Age**								
40–49	9.4	+/−0.1	10.7	+/−0.1	59.9	+/−0.5	62.7	+/−0.4
50–59	9.5	+/−0.1	10.4	+/−0.1	61.7	+/−0.5	63.7	+/−0.4
60–69	9.0	+/−0.1	9.5	+/−0.1	61.4	+/−0.5	63.2	+/−0.5
70–79	8.6	+/−0.2	9.4	+/−0.2	63.8	+/−0.8	66.3	+/−0.6
**Education**								
<9 years	8.5	+/−0.2	8.8	+/−0.1	60.0	+/−0.7	63.0	+/−0.6
9–11 years	8.6	+/−0.1	9.4	+/−0.1	58.2	+/−0.6	61.3	+/−0.5
High school	9.1	+/−0.1	10.0	+/−0.1	60.6	+/−0.5	62.8	+/−0.4
Some college/junior college	9.7	+/−0.1	11.0	+/−0.1	63.2	+/−0.5	65.2	+/−0.5
College degree and beyond	9.7	+/−0.1	10.9	+/−0.1	66.6	+/−0.6	67.5	+/−0.5
**Household Income ($)**								
<15 K	8.9	+/−0.1	9.3	+/−0.1	61.4	+/−0.5	62.6	+/−0.4
15–24.9 K	8.9	+/−0.1	9.8	+/−0.1	59.5	+/−0.5	62.0	+/−0.4
25–49.9 K	9.2	+/−0.1	10.4	+/−0.1	60.9	+/−0.6	64.3	+/−0.5
≥50 K	9.5	+/−0.2	10.5	+/−0.2	64.9	+/−0.7	67.0	+/−0.7
**Currently Working**								
Yes	9.3	+/−0.1	10.2	+/−0.1	56.9	+/−0.5	59.7	+/−0.4
No	9.0	+/−0.1	9.8	+/−0.1	66.5	+/−0.5	68.2	+/−0.4
**Body Mass Index (kg/m** 2 **)**								
<18.5	8.7	+/−0.3	10.0	+/−0.3	58.9	+/−1.3	63.8	+/−1.1
18.5–24.9	8.4	+/−0.1	9.7	+/−0.1	59.2	+/−0.5	63.2	+/−0.4
25–29.9	8.9	+/−0.1	9.9	+/−0.1	61.3	+/−0.5	63.1	+/−0.4
30–34.9	9.3	+/−0.1	10.0	+/−0.1	62.7	+/−0.5	64.0	+/−0.4
35+	10.2	+/−0.1	10.4	+/−0.1	66.5	+/−0.5	65.8	+/−0.4
**Smoking Status**								
Current	9.5	+/−0.1	10.7	+/−0.1	61.9	+/−0.5	64.5	+/−0.4
Former	9.1	+/−0.1	9.9	+/−0.1	62.0	+/−0.5	64.3	+/−0.4
Never	8.8	+/−0.1	9.5	+/−0.1	61.2	+/−0.5	63.1	+/−0.4
**Marital Status**								
Married	8.8	+/−0.1	9.8	+/−0.1	59.0	+/−0.5	61.9	+/−0.4
Separated/Divorced	9.2	+/−0.1	10.2	+/−0.1	62.6	+/−0.5	64.4	+/−0.4
Single (Never married)	9.4	+/−0.2	10.1	+/−0.1	63.2	+/−0.7	64.7	+/−0.5
Widowed	9.1	+/−0.1	9.9	+/−0.1	61.9	+/−0.6	64.9	+/−0.5

1 Includes total/occupational activity plus sports/exercise.

2 Wake time includes sitting, household/occupational activity, and sports.

NOTE: All models are adjusted for the other demographic characteristics shown in the table as well as for hypertension (Y/N), diabetes (Y/N), heart attack/coronary artery disease (Y/N), stroke/TIA (Y/N), and emphysema (Y/N).

### Association of Active Behaviors and Demographics

Summary measures of physically active behaviors showed relatively consistent patterns in blacks and whites (Tables 6 and 7). Age was inversely associated with all three overall physical activity indices. Total physical activity (MET-hrs/day) tended to be lower among those with the highest levels of education and income while the adjusted percentage of participants meeting the current physical activity recommendations (via sports and exercise) rose monotonically with rising education and income. Individuals who were currently working reported substantially higher levels of total physical activity, and they also reported a higher prevalence of meeting current physical activity recommendations. No clear patterns of activity were seen among males in relation to marital status, but among women, total household/occupational activity and total activity overall were both higher among married women than among those who were divorced, widowed, or single.

**Table 6:** Physical activity behaviors among males [adjusted mean (std err) or frequency] across demographic characteristics and body mass index, by race.

	Total household/occupational activity (MET-hrs/d)				Overall activity (MET-hrs/d)^1^				Meets current physical activity recommendations via sports/exercise (%)^2^			
	White		Black		White		Black		White		Black	
**Age**												
40–49	23.1	+/−0.7	22.0	+/−0.5	24.5	+/−0.7	23.7	+/−0.5	24.4	+/−1.5	26.4	+/−1.1
50–59	19.5	+/−0.7	17.7	+/−0.5	20.6	+/−0.7	18.8	+/−0.5	18.2	+/−1.5	18.8	+/−1.1
60–69	18.1	+/−0.8	14.3	+/−0.6	19.1	+/−0.8	15.4	+/−0.6	19.5	+/−1.6	17.1	+/−1.3
70–79	16.5	+/−1.0	13.3	+/−0.9	17.2	+/−1.1	14.1	+/−0.9	16.7	+/−2.2	13.5	+/−1.9
**Education**												
<9 years	20.8	+/−1.0	15.5	+/−0.7	21.5	+/−1.0	16.3	+/−0.7	13.5	+/−2.1	12.0	+/−1.4
9–11 years	21.5	+/−0.8	18.3	+/−0.6	22.5	+/−0.9	19.3	+/−0.6	17.4	+/−1.8	15.8	+/−1.2
High school	22.1	+/−0.7	18.5	+/−0.5	23.1	+/−0.7	19.6	+/−0.6	17.9	+/−1.5	19.0	+/−1.2
Some college/junior college	19.3	+/−0.8	17.7	+/−0.6	20.5	+/−0.8	19.1	+/−0.6	22.0	+/−1.6	22.2	+/−1.2
College degree and beyond	12.7	+/−0.8	14.2	+/−0.7	14.3	+/−0.8	15.7	+/−0.7	27.7	+/−1.7	25.6	+/−1.4
**Household Income ($)**												
<15 K	16.7	+/−0.7	17.1	+/−0.5	17.2	+/−0.7	17.9	+/−0.5	11.7	+/−1.5	13.0	+/−1.1
15–24.9 K	21.8	+/−0.8	18.7	+/−0.5	22.8	+/−0.8	19.8	+/−0.6	16.0	+/−1.7	17.7	+/−1.2
25–49.9 K	21.0	+/−0.8	18.3	+/−0.6	22.1	+/−0.9	19.6	+/−0.6	19.8	+/−1.8	21.0	+/−1.3
≥50 K	17.6	+/−0.9	13.2	+/−0.8	19.3	+/−0.9	14.7	+/−0.8	31.2	+/−1.9	24.0	+/−1.6
**Currently Working**												
Yes	25.7	+/−0.7	25.0	+/−0.5	26.7	+/−0.8	26.3	+/−0.6	20.9	+/−1.6	21.5	+/−1.1
No	12.9	+/−0.7	8.6	+/−0.5	14.0	+/−0.7	9.7	+/−0.5	18.5	+/−1.4	16.3	+/−1.1
**Body Mass Index (kg/m** 2 **)**												
<18.5	21.1	+/−2.2	12.8	+/−1.3	21.6	+/−2.3	13.6	+/−1.3	14.4	+/−4.7	12.7	+/−2.7
18.5–24.9	20.5	+/−0.6	18.3	+/−0.5	22.1	+/−0.6	19.6	+/−0.5	25.4	+/−1.3	21.7	+/−1.1
25–29.9	19.4	+/−0.6	18.6	+/−0.5	20.7	+/−0.6	20.0	+/−0.5	23.2	+/−1.2	23.5	+/−1.0
30–34.9	18.6	+/−0.6	17.8	+/−0.5	19.7	+/−0.7	19.0	+/−0.5	19.2	+/−1.4	20.7	+/−1.1
35+	16.8	+/−0.7	16.7	+/−0.6	17.7	+/−0.8	17.7	+/−0.6	16.3	+/−1.6	16.1	+/−1.3
**Smoking Status**												
Current	21.5	+/−0.7	18.5	+/−0.5	22.4	+/−0.7	19.5	+/−0.6	15.0	+/−1.5	18.2	+/−1.1
Former	18.5	+/−0.7	16.1	+/−0.5	19.7	+/−0.7	17.4	+/−0.6	21.4	+/−1.5	19.5	+/−1.2
Never	17.9	+/−0.8	15.9	+/−0.6	19.0	+/−0.8	17.1	+/−0.6	22.8	+/−1.6	19.1	+/−1.2
**Marital Status**												
Married	18.1	+/−0.6	16.9	+/−0.5	18.8	+/−0.6	17.8	+/−0.5	16.0	+/−1.3	16.1	+/−1.1
Separated/Divorced	20.2	+/−0.7	17.2	+/−0.5	21.4	+/−0.7	18.5	+/−0.5	21.6	+/−1.5	20.0	+/−1.1
Single (Never married)	18.7	+/−0.8	16.9	+/−0.6	19.9	+/−0.9	18.2	+/−0.6	20.6	+/−2.6	18.9	+/−1.8
Widowed	20.1	+/−1.2	16.3	+/−0.8	21.3	+/−1.3	17.5	+/−0.9	20.5	+/−1.8	20.6	+/−1.2

1 Includes household/occupational activity and sports/exercise.

2 Current recommendations are met if participant reported 150+ min/week of moderate sports/exercise or 75+ min/week of vigorous sports/exercise.

NOTE: All models are adjusted for the other demographic characteristics shown in the table as well as for hypertension (Y/N), diabetes (Y/N), heart attack/coronary artery disease (Y/N), stroke/transient ischemic attack (Y/N), and emphysema (Y/N).

**Table 7:** Physical activity behaviors among females [adjusted mean (std err) or frequency] across demographic characteristics and body mass index, by race.

	Total household/occupational activity (MET-hrs/d)				Overall activity (MET-hrs/d)^1^				Meets current physical activity recommendations via sports/exercise (%)^2^			
	White		Black		White		Black		White		Black	
**Age**												
40–49	20.9	+/−0.4	20.5	+/−0.3	22.3	+/−0.4	21.6	+/−0.3	22.4	+/−0.9	19.3	+/−0.8
50–59	19.3	+/−0.4	19.1	+/−0.3	20.4	+/−0.4	20.0	+/−0.3	18.0	+/−0.9	15.7	+/−0.8
60–69	18.1	+/−0.4	17.4	+/−0.4	19.1	+/−0.4	18.3	+/−0.4	17.8	+/−1.0	14.3	+/−0.9
70–79	15.9	+/−0.6	15.6	+/−0.5	16.7	+/−0.6	16.3	+/−0.5	17.0	+/−1.4	11.9	+/−1.2
**Education**												
<9 years	19.0	+/−0.6	17.2	+/−0.4	19.8	+/−0.6	17.8	+/−0.5	13.8	+/−1.4	9.9	+/−1.1
9–11 years	21.3	+/−0.4	19.1	+/−0.4	22.2	+/−0.5	19.8	+/−0.4	15.1	+/−1.1	13.1	+/−0.9
High school	19.7	+/−0.4	19.2	+/−0.3	20.7	+/−0.4	20.0	+/−0.3	17.2	+/−0.9	14.5	+/−0.8
Some college/junior college	18.1	+/−0.4	18.8	+/−0.3	19.3	+/−0.4	19.9	+/−0.4	21.5	+/−1.0	18.1	+/−0.8
College degree and beyond	14.7	+/−0.4	16.4	+/−0.4	16.2	+/−0.4	17.7	+/−0.4	26.5	+/−1.0	21.0	+/−0.9
**Household Income ($)**												
<15 K	18.8	+/−0.3	18.2	+/−0.3	19.6	+/−0.4	18.8	+/−0.3	13.8	+/−0.8	11.3	+/−0.7
15–24.9 K	20.4	+/−0.4	19.9	+/−0.3	21.2	+/−0.4	20.6	+/−0.3	15.5	+/−1.0	13.7	+/−0.8
25–49.9 K	19.3	+/−0.4	18.3	+/−0.4	20.4	+/−0.5	19.3	+/−0.4	18.4	+/−1.1	16.4	+/−0.9
≥50 K	15.8	+/−0.5	16.4	+/−0.5	17.3	+/−0.5	17.5	+/−0.5	27.5	+/−1.2	19.8	+/−1.2
**Currently Working**												
Yes	23.3	+/−0.4	22.3	+/−0.3	24.4	+/−0.4	23.2	+/−0.3	20.1	+/−0.9	16.0	+/−0.8
No	13.9	+/−0.3	14.0	+/−0.3	14.9	+/−0.4	14.9	+/−0.3	17.5	+/−0.8	14.6	+/−0.7
**Body Mass Index (kg/m** 2 **)**												
<18.5	20.0	+/−1.0	18.6	+/−0.9	21.7	+/−1.0	19.7	+/−0.9	24.9	+/−2.3	16.5	+/−2.1
18.5–24.9	19.5	+/−0.4	18.5	+/−0.3	20.7	+/−0.4	19.5	+/−0.3	23.2	+/−0.9	18.0	+/−0.8
25–29.9	18.5	+/−0.3	18.4	+/−0.3	19.5	+/−0.4	19.3	+/−0.3	17.8	+/−0.8	16.7	+/−0.7
30–34.9	18.2	+/−0.4	18.1	+/−0.3	18.9	+/−0.4	18.9	+/−0.3	15.1	+/−0.9	14.1	+/−0.7
35+	16.6	+/−0.4	17.3	+/−0.3	17.3	+/−0.4	17.8	+/−0.3	13.0	+/−0.9	11.3	+/−0.7
**Smoking Status**												
Current	19.3	+/−0.4	18.9	+/−0.3	20.2	+/−0.4	19.7	+/−0.3	15.7	+/−0.9	14.4	+/−0.8
Former	18.1	+/−0.4	17.8	+/−0.3	19.3	+/−0.4	18.7	+/−0.4	20.9	+/−0.9	16.3	+/−0.8
Never	18.3	+/−0.4	17.9	+/−0.3	19.4	+/−0.4	18.7	+/−0.3	19.8	+/−0.9	15.3	+/−0.8
**Marital Status**												
Married	19.8	+/−0.3	19.2	+/−0.3	20.8	+/−0.4	20.0	+/−0.3	17.2	+/−0.8	14.1	+/−0.8
Separated/Divorced	18.2	+/−0.4	18.2	+/−0.3	19.3	+/−0.4	19.1	+/−0.3	18.6	+/−0.9	15.8	+/−0.8
Single (Never married)	17.8	+/−0.5	17.8	+/−0.4	18.9	+/−0.6	18.7	+/−0.4	20.3	+/−1.1	15.2	+/−0.9
Widowed	18.4	+/−0.4	17.4	+/−0.4	19.5	+/−0.5	18.3	+/−0.4	19.1	+/−1.3	16.1	+/−0.9

1 Includes household/occupational activity and sports/exercise.

2 Current recommendations are met if participant reported 150+ min/week of moderate sports/exercise or 75+ min/week of vigorous sports/exercise.

NOTE: All models are adjusted for the other demographic characteristics shown in the table as well as for hypertension (Y/N), diabetes (Y/N), heart attack/coronary artery disease (Y/N), stroke/transient ischemic attack (Y/N), and emphysema (Y/N).

## Discussion

In this large cohort in the southeastern United States, we found that both black and white middle age and older men and women reported durations and patterns of sedentary and physically active behaviors that were strikingly similar to one another. Thus, it seems likely that physical activity patterns do not play a large role in the racial disparities observed in the incidence of chronic diseases, health outcomes, or mortality in our study population.

This study adds important new information related to sedentary behaviors in black and white adults. Sedentary behaviors have emerged as a potentially important component of overall health because of their relationship to diseases such as metabolic syndrome [7] and diabetes [24] as well as overall mortality [6], [25] independent of physical activity. We found that adults in the SCCS spent about 60% of their time in sedentary pursuits, a finding similar to that observed in an examination of 1,640 black and 2,747 white participants in the 2003–2004 National Health and Nutrition Examination Survey (NHANES) who wore activity monitors [26]. While television and movie viewing was the single largest sedentary behavior in the SCCS, it only accounted for about half of all sedentary time reported. Blacks reported spending about 20 minutes per day (2.2%) more time in sedentary behaviors than whites, a difference largely due to increased television viewing among blacks. This finding is consistent with other reports showing that blacks spend more time viewing television than whites [27], [28]. Our finding of higher sedentary time in blacks compared with whites (45 minutes for men and 15 minutes for women) differs, however, from NHANES data in which no racial differences in sedentary behaviors were observed in women, while white men age 40–59 had approximately 15 minutes more sedentary time per day than black men of the same age (with no racial differences seen in men older than 60) [26]. This could be explained by residual confounding by SES in the nationally representative sample in NHANES compared with the SCCS, regional differences between the NHANES and SCCS populations, or by differences in methodology for ascertaining sedentary behaviors (activity monitors versus questionnaire).

Similar levels of total physical activity (which includes household and occupational activity as well as sports and exercise) were observed in our study for black and white adults, and these results are generally consistent with a number of studies that have also examined either occupational [11], [12], household [11], or total physical activity levels [29], [30] across race groups. Evenson and colleagues examined data from the Atherosclerosis Risk in Communities (ARIC) Study and compared 2,991 black to 8,566 white adults on their occupational activity levels using the Baecke questionnaire. This study found higher levels of occupational activity among blacks but higher leisure activity among whites, indicating that differences in total physical activity may not differ greatly by race when all domains are considered [12]. Troiano and colleagues recently evaluated objectively determined physical activity levels using NHANES data and observed no differences in overall activity levels between black and white men or women [29]. The collective results from these studies suggest that total physical activity levels may not differ by race, and moreover, differences in the prevalence of leisure-time activity participation between blacks and whites derived from national surveillance systems [10], [13] do not appear to translate into differences in total physical activity levels.

In this study, the proportions of adults meeting current physical activity recommendations via sports/exercise were similar between black and white males as well as black and white females. Overall, only 16% of women and 25% of men met the current physical activity recommendations via sports and exercise. Our results are consistent with 2009 Behavioral Risk Factor Surveillance Study (BRFSS) data showing that adults in southeastern states have lower levels of moderate and vigorous physical activity than in other regions of the US [31]. The similarities observed between race groups in the SCCS provide some contrast to earlier studies showing that black adults had lower participation in leisure-time physical activity than whites [10]–[13], [32]. According to 2008 NHIS data, the prevalence of being aerobically active in line with the *2008 Physical Activity Guidelines for Americans* was lower for black compared to white adults (33.9% versus 47.6%) [13].

There was marked variation by education and income, with 10–14% of those in the lowest versus 21–28% in the highest levels meeting the physical activity recommendations among both blacks and whites. Interestingly, the most educated, and often the most affluent, participants in our study also reported the lowest levels of overall activity and the most time spent in sedentary behaviors. These results are perhaps reflective of the types of jobs held by highly educated individuals that require long hours or extended periods of sitting at a desk or working on a computer. Crespo and colleagues evaluated the role of differences in SES factors to explain previously reported physical activity differences by race, but found that adjustment for a variety of SES indicators did not entirely account for observed disparities in physical activity participation [10]. One potential explanation for the differences between our findings of similar proportions of sedentary time, total physical activity, and meeting the current physical activity recommendations in black and white adults and differences reported by other national studies is that, by design, SCCS participants of both races were of similar SES and lived in the same communities, reducing the potential for SES-related confounding. Indeed, the marked differences by SES within the SCCS suggest that even moderate uncontrolled confounding by SES could create the appearance of a racial difference.

Ford et al. observed similar patterns to ours regarding physical activity behaviors and SES in a study of lower and higher income adults living in Pittsburg, Pennsylvania, United States [30]. Their study found differences between men and women and among the SES groups independent of race (white vs. black). In women, the total amount of physical activity was similar in lower and higher SES groups; however, women with lower SES were less likely to engage in leisure time physical activity, but more likely to engage in walking for transport and household activities. In men, total physical activity was nearly identical between the SES groups but men from the lower SES group spent significantly more time each week walking and doing household chores, whereas men from the higher SES group tended to be more active in leisure-time physical activity, independent of race.

In this context, our findings underscore the earlier work by Ford et al. [30] in suggesting that racial disparities in both sedentary and physically active behaviors may be minimized when SES indicators are carefully controlled either by study design or by the collection of extensive confounder data that are used in the analysis.

Our study has several strengths. First, it is one of the largest studies to date to evaluate patterns of physical activity-related behavior in black and white adults with a substantial representation of low-income participants. First, although the SCCS population is not reflective of the socioeconomic or racial distributions in the general population of the US or the southeast in particular due to the recruitment strategy through CHCs and the resulting over-representation of low-income individuals, it is a uniquely designed cohort in which to study health effects in blacks compared with whites both because of the large number of blacks and the comparability of socioeconomic status between the racial groups. Thus, this study design reduced the potential for unmeasured confounding due to a number of important SES factors, community level factors, and region of the country. In our analyses, we also controlled for and evaluated the effect of a number of demographic and health factors. An additional strength of this research was that we assessed our behavioral outcomes via in-person interview using an instrument that we designed to evaluate a broad range of activity behaviors. Therefore we were able to evaluate participants on their overall levels of these behaviors, rather than only examining a few index behaviors, such as leisure-time activity and television viewing. Finally, our PAQ included several questions that assessed time spent in sedentary behaviors which has recently emerged as an important component of overall daily activity.

Limitations of this study should also be considered. First, all information on activities was self-reported. Since an important objective of this study was to compare levels of active and sedentary behaviors by race, differential reporting bias by race is a particular concern. Walsh and colleagues compared self-reports of physical activity in black and white premenopausal women and found that while black and white women reported similar levels of activity, and misreported to a similar degree, following a weight loss intervention, black women over-reported their activity levels to a greater extent than their white counterparts [33]. Little is known about the potential for differential reporting by race among men, but accuracy of physical activity questionnaire measurements was found to be higher overall in men (compared with women) in at least one study [34]. Differential reporting by body size has also been reported with overweight and obese individuals over-reporting physical activity more so than those of a healthy body weight [34]–[36], a concern given the much higher prevalence of obesity among black women compared with white women in the SCCS. Overall, however, the general consistency of our findings with national data derived from objectively determined activity [26], [29] argues against differential reporting biases exerting a major influence on our results. An additional limitation is that we were unable to evaluate other potential determinants of physical activity level, particularly as they relate to psychological factors associated with participation and adherence in physical activities or the local environments in which our participants reside. However, to the extent that the Community Health Centers from which our population was recruited drew from all communities and neighborhoods nearest them, both black and white participants were likely to have been exposed to similar environmental conditions. A final limitation is that our study sample includes both participants who enrolled in-person at CHCs (86% of participants) and those who responded to a paper questionnaire sent to a random sample of the general population in same states as the CHC enrollment (14% of the participants). Differential response patterns to the PAQ could have arisen due to the differing modes of data collection (in-person versus paper questionnaire) or due to the underlying differences in the two groups (with the general population group having higher education levels and household income than the CHC-enrolled participants) although this was ameliorated somewhat in our multivariate models by adjusting for education and income.

In conclusion, these data provide unique insights into the descriptive epidemiology of active and sedentary behaviors in a large group of predominantly low-income middle-age and older adults residing in the southeastern United States, an area at particular risk for many chronic diseases that may be related to physical activity. The observed predominance of sedentary behaviors and low amount of moderate and vigorous physical activity in the daily life of the SCCS participants indicate that future physical activity guidelines should focus not only on increasing high intensity activities such sports and exercise but also on reducing sedentary behaviors in all race and gender groups.
